# Spread of pathology in amyotrophic lateral sclerosis: assessment of phosphorylated TDP-43 along axonal pathways

**DOI:** 10.1186/s40478-015-0226-y

**Published:** 2015-07-28

**Authors:** Manaal Fatima, Rachel Tan, Glenda M. Halliday, Jillian J. Kril

**Affiliations:** Discipline of Pathology, University of Sydney, Level 6 W Charles Perkins Centre D17, Sydney, 2006 Australia; School of Medical Sciences, Faculty of Medicine, University of New South Wales, Sydney, 2052 Australia; Neuroscience Research Australia, Sydney, 2031 Australia; Discipline of Medicine, University of Sydney, Sydney, 2006 Australia

**Keywords:** Amyotrophic lateral sclerosis, Disease staging, Oligodendrocyte inclusions, pTDP-43

## Abstract

**Introduction:**

The progression of amyotrophic lateral sclerosis (ALS) through the brain has recently been staged using independent neuropathological and neuroimaging modalities. The two schemes tie into the concept of pathological spread through corticofugal axonal transmission that stems from observation of oligodendrocyte pTDP-43 aggregates along with neuronal inclusions. Here, we aimed to assess evidence of transmission along axonal pathways by looking for pTDP-43 oligodendrocyte pathology in involved white matter tracts, and to present a first validation of the neuropathological staging scheme.

pTDP-43 immunohistochemistry was performed in select white matter tracts and grey matter regions from the staging scheme in postmortem-confirmed ALS cases (*N* = 34). Double-labelling immunofluorescence was performed to confirm co-localisation of pTDP-43 immunoreactivity to oligodendrocytes.

**Results:**

While pTDP-43 immunoreactive oligodendrocytes were frequent in the white matter under the motor and sensory cortices, similar assessment of the white matter along the corticospinal tract and in the corpus callosum and cingulum bundle of the same cases revealed no pTDP-43 pathology, questioning the involvement of oligodendrocytes in pathological propagation. The assessment of Betz cell loss revealed that the lack of deep white matter pTDP-43 oligodendrocyte pathology was not due to an absence of motor axons. Assessment of the propagation of pathology to different grey matter regions validated that all cases could be allocated to one of four neuropathological stages, although Stage 4 cases were found to differ significantly in age of onset (~10 years older) and disease duration (shorter duration than Stage 3 and similar to Stage 2).

**Conclusions:**

Four stages of ALS neuropathology can be consistently identified, although evidence of sequential clinical progression requires further assessment. As limited pTDP-43 oligodendrocyte pathology in deep corticospinal and other white matter tracts from the motor cortex was observed, the propagation of pathology between neurons may not involve oligodendrocytes and the interpretation of the changes observed on neuroimaging should be modified accordingly.

**Electronic supplementary material:**

The online version of this article (doi:10.1186/s40478-015-0226-y) contains supplementary material, which is available to authorized users.

## Introduction

Motor neuron disease covers a group of neurodegenerative disorders that affect different parts of the motor system, be it the upper motor neurons in the primary motor cortex and brainstem or the lower motor neurons in the spinal cord. Amyotrophic lateral sclerosis (ALS) with both upper and lower motor neuron involvement is the most common presentation, accounting for more than 75 % of cases with motor neuron disease [1, 2]. The key histological feature of sporadic ALS is related to the formation of cytoplasmic inclusions containing phosphorylated 43-kDA TAR DNA-binding protein (pTDP-43) [3, 4]. Although the motor system is primarily affected in ALS, a large body of neuropathological and neuroimaging evidence suggests that non-motor regions become progressively involved [5–10].

Recently, Brettschneider and colleagues defined four neuropathological stages of ALS [6], proposing that lesions first develop in the motor cortex, brainstem motor nuclei of cranial nerves V, VII and X-XII, and spinal cord α-motoneurons in Stage 1. In Stage 2, the prefrontal cortex, reticular formation, precerebellar nuclei and red nucleus are seemingly involved, followed by further expansion into the prefrontal and postcentral cortices and striatum by Stage 3. Finally, the pathology is thought to extend to anteromedial portions of the temporal lobe and eventually affect the hippocampus by Stage 4. The earliest and most common lesions are found in oligodendrocytes [6], and in association with animal studies, it has been proposed that early oligodendrocyte involvement is important for the propagation of pathology to neurons in ALS [7, 11–14].

The idea of disease propagation from one brain region to the next has also been reinforced by cell models [15] and neuroimaging studies [9, 10]. In assessing white matter tract involvement, Kassubek and colleagues were able to differentiate ALS patients into four similarly staged groups [8], with corticospinal tract involvement in Stage 1, corticorubral and corticopontine involvement in Stage 2, corticostriatal in Stage 3 and the perforant pathway involved in Stage 4. A positive correlation between these ALS stages and disease duration was found, with an average increase in disease duration from 10 months at Stage 1 to 20 months at Stage 4 in the 88 cases analysed [8]. While there are limited longitudinal studies [9, 10], it seems that grey matter is more affected over time compared to the severity and spread of white matter changes [16], although further longitudinal studies are needed to validate cross-sectional data concerning potential disease progression.

While white matter involvement is a prominent feature in neuroimaging investigations of the ALS brain and substantiates the idea of spread along these tracts, to our knowledge no study has tried to verify this concept by looking for the presence of pathology in the core white matter regions implicated. The primary objective of this investigation was to identify pathological evidence of transmission along axonal pathways by looking for pTDP-43 oligodendrocyte pathology in regions implicated by neuroimaging, namely the corticospinal tract in the posterior limb of the internal capsule, the corpus callosum and the cingulum bundle [8]. We also present the first independent validation of the neuropathological staging scheme proposed by Brettschneider and colleagues [6].

## Material and methods

### Cases

ALS cases were collected by the New South Wales Tissue Resource Centre with approval from the Human Ethics Committee of the University of Sydney. Inclusion criteria were neurologically and neuropathologically confirmed ALS with pTDP-43 pathology [17]. Exclusion criteria were strong family history of a neurological condition, presence of co-existing dementia or neuropathology sufficient to reach criteria for another neurodegenerative disease [18, 19]. Thirty-four ALS patients met criteria (*n* = 21 males, *n* = 13 females, age range = 47-81 years) and were matched for age and sex to controls with no evidence of a neurological or neuropathological disease (*n* = 3 males, *n* = 2 females, age range = 55-80 years). ALS cases had variable deficits at disease onset, including upper limb onset (*n* = 11), lower limb onset (*n* = 14) and bulbar onset (*n* = 9), but these subsets did not differ in age at onset, age at death or disease duration (Table 1). Ten ALS cases had the presence of a C9ORF72 expansion confirmed on DNA testing or inferred through staining of dipeptide repeats in the cerebellum [20]. This study was approved by the Human Research Ethics Committees of the University of New South Wales and the University of Sydney.

**Table 1:** Case demographics^a^ and clinical details

	Control	All ALS	ALS Subtype		
Presenting deficit	-	-	Upper limb	Lower limb	Bulbar
N	5	34	11	14	9
M:F	3:2	21:13	6:5	11:3	4:5
Mean age at death, y (SD)	67 (10)	64 (8)	65 (10)	62 (9)	66 (7)
Range, y	55-80	47-81	47-81	53-77	57-78
Mean duration, y (SD)	-	2 (2)	3 (2)	2 (1)	3 (2)
Range, y	-	0.4-7	1-3.6	0.4-6.1	0.6-4.5
Mean PMI, h (SD)	22 (13)	21 (19)	18 (18)	19 (7)	26 (29)
Range, h	12-43	5-99	5-72	7-31	5-99

^a^No differences between groups

### Tissue sampling and staining

To look for evidence of pathological spread, de-identified formalin-fixed, paraffin embedded tissue blocks were obtained and sectioned at 10 μm. Fixed brain tissue in 19 cases (56 %) was taken from the hemisphere contralateral to the side first affected clinically, in 4 cases (12 %) the sampled region was ipsilateral to the first affected side and in 11 cases (32 %) laterality of onset was bilateral or unknown. In all but three cases, validation of grey matter disease staging was performed using the following recommended blocks [6]: (1) the primary motor cortex with the underlying white matter (affected in Stage 1), (2) the medulla oblongata taken at the level of nerve XII containing the reticular formation and inferior olive (lower motor neurons affected in Stage 2), (3) the sensory cortex of the post-central gyrus (affected in Stage 3,), and (4) the hippocampal formation (affected in Stage 4). In the event that no pathology was observed in the sensory cortex for Stage 3, (5) the striatum (caudate nucleus and putamen) was examined as an alternate region involved in Stage 3 [6].

The following regions implicated in the neuroimaging literature [8–10] were sampled to assess oligodendroglia spreading of pathological TDP-43: (A) the corticospinal tract in the posterior limb of the internal capsule, (B) corpus callosum sampled at the level of the mammillary bodies, and (C) cingulum bundle also sampled at the level of the mammillary bodies. Three adjacent sections of these regions were cut in five cases with the most severe pathology in the motor cortex to ensure immunopositive oligodendrocytes were not missed due to the use of 10 μm sections. ALS neuropathology was identified using immunoperoxidase staining with antibodies to pTDP-43 (pS409/410, 1:40,000, Cosmo Bio) as well as native TDP-43 (1:1000, Protein Tech) in a subset of five cases, both optimised following the manufacturer’s standard protocol. Antigen retrieval was performed by boiling for 25 min in 0.01 M sodium citrate buffer solution (pH 6.0) for the pTDP-43 antibody and Tris EDTA (pH 9.0) buffer solution for the native TDP-43 antibody. The primary antibodies were incubated for 48 h at 4 °C and for 1 h at 37 °C respectively, and visualised using secondary EnVision Dual Link Polymer (Dako) and DAB (Sigma-Aldrich), followed by a haematoxylin counterstain.

### Assessment of ALS neuropathology

Validation of the ALS neuropathological staging scheme was performed in all but three cases by assessing the presence (+) or absence (−) of pTDP-43 pathology in the regions listed above. Based on the regional involvement outlined in Table 2, each case was assigned to one of the four neuropathological stages. In the remaining three cases, pTDP-43 pathology observed in the hippocampus was consistent with Stage 4 ALS.

**Table 2:** Criteria for stage allocation and distribution of cases^a^

	Motor cortex	Brainstem	Sensory cortex/ striatum	Hippocampus	Number of cases
Stage 1	+	-	-	-	1 (3 %)
Stage 2	+	+	-	-	8 (24 %)
Stage 3	+	+	+	-	16 (47 %)
Stage 4^a^	+	+	+	+	9 (26 %)

^a^ Note three cases had pTDP-43 pathology observed in the hippocampus consistent with Stage 4 ALS

The severity of Betz cell loss and pTDP-43 pathology (including neuronal cytoplasmic inclusions, glial cytoplasmic inclusions and dystrophic neurites) was also evaluated. For Betz cell loss, the motor cortex from 32 the cases was assessed in sections taken from 3–5 consecutive blocks by an experienced neuropathologist, and the number of healthy-looking, normal-sized Betz cells in the lower half of the motor strip in each section was graded from 0 (no Betz cells) to 3 (number of Betz cells typically seen in a healthy subject). The scores from each section were averaged to produce a final grade for each case.

For pTDP-43 severity, the densest region of pTDP-43 pathology in the grey and white matter of 31 cases was independently graded under a 20x objective using a semi-quantitative scoring system: 0- pathology absent; 1- sparse pathology with no more than 5 inclusions in the entire region; 2- mild pathology with on average 1–5 inclusions in the densest region; 3- moderate pathology with an average of 6–10 inclusions in the densest region; 4- severe pathology with an average of 11 or more inclusions in the densest region (Additional file 1: Figure S1). Assessment of the inter-rater agreement on the severity of pTDP-43 pathology grading in ten randomly selected cases using κ statistics revealed near perfect agreement (κ = 0.8) [21].

### Assessment of oligodendroglial involvement

Double-labelling immunofluorescence was performed using antibodies for ALS neuropathology (pTDP-43; 1:10,000) and oligodendroglia (anti-tubulin polymerisation promoting protein p25α, Abcam; 1:250) [22] to confirm localisation of pathology in non-neuronal cells. Antigen retrieval was performed as outlined above and the primary antibody cocktail was incubated for 48 h at 4 °C and visualised using an anti-mouse secondary antibody conjugated to Alexa 594 (1:200) and anti-rabbit secondary antibody conjugated to Alexa 488 (1:200) (Molecular Probes). Slides were coverslipped with ProLong Gold Antifade Reagent with DAPI (Molecular Probes) and assessed using optional sectioning with the Apotome.2 on the Zeiss AxioImager.M2. Specificity of immunofluorescence was tested by: 1) excluding one of the primary antibodies (only appropriate single labelling was observed) and 2) excluding both primary antibodies (no immunofluorescence observed). The motor cortex (highest density of ALS neuropathology) in the six most severe ALS cases was chosen for evaluation. Ten images of grey matter and ten images of white matter were taken at 200x magnification (approximately 8 % of the average motor strip) and the number of inclusions observed in neuronal cell bodies (identified by their nucleoli), oligodendrocytes (identified by p25α immunoreactivity) or “other” structures were counted and averaged across all images to produce a representative distribution of pTDP-43 co-localisation across different cell types. “Other” structures included pTDP-43 immunoreactivity in non-p25α-immunoreactive glial cells with an obvious nucleus, potential p25α immunoreactive pTDP-43 extra-axonal inclusions (not associated with a nucleus), or pTDP-43 inclusions detached from any other identifiable structures, such as dystrophic neurites. Assessment of the inter-rater agreement in the number of pTDP-43-immunoreactive oligodendrocyte inclusions in the images (*N* = 10 images compared out of 60 available) revealed <8 % difference between two investigators.

### Statistics

All statistical analysis was performed using SPSS software (version 21 for Mac, SPSS Inc., Chicago, IL, USA). Means and standard deviations are shown. Kruskal-Wallis and Mann–Whitney U statistics were used to determine differences between groups and Spearman rank correlations and *χ*^2^ analyses performed to determine relationships between clinical and pathological variables. *p* <0.05 was considered statistically significant.

## Results

### Pathological evidence of transmission along axonal pathways

**Fig. 1:**
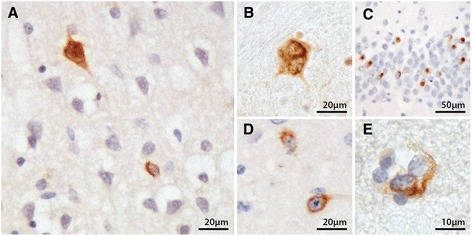
pTDP-43 pathology in the motor cortex (**a**), inferior olivary nucleus (**b**), hippocampal dentate gyrus (**c**), caudate nucleus (**d**), and oligodendrocytes in the white matter underlying the motor cortex (**e**)

As expected, all ALS cases were immunopositive for pTDP-43 pathology in one or more of the grey matter regions examined: i.e. the motor cortex, brainstem, sensory cortex, striatum and hippocampus (see Fig. 1). As expected, no pathology was observed in any of the controls. Surprisingly, assessment of the posterior limb of the internal capsule, the corpus callosum and the cingulum bundle revealed no pTDP-43 pathology. Inclusions were absent from sections stained with both the phosphorylated and native TDP-43 antibodies, even when serial sections were examined. The only white matter pTDP-43 observed in the samples was in the subcortical white matter associated with the motor and sensory cortices. The pTDP-43 white matter pathology observed in these regions was largely associated with oligodendrocytes (see Fig. 1e). The absence of pTDP-43 pathology further along these axonal tracts was an unexpected finding, particularly considering the large body of evidence to suggest white matter alterations in these regions [8–10], and also because the selected regions sampled from the internal capsule, corpus callosum and cingulum bundle were closely associated with the motor network. Instead of the expected oligodendroglial transmission along axonal pathways, the observation of pTDP-43 immunoreactivity in oligodendrocytes close to cortical areas suggests a more localised mechanism of oligodendroglial involvement within regions of close proximity.

### Grey matter pTDP-43 pathology and validation of the ALS staging scheme in a representative cohort

In the motor cortex, pTDP-43 immunopositive neuronal cytoplasmic inclusions (Fig. 1a), glial cytoplasmic inclusions and dystrophic neurites were identified. In the brainstem, immunopositive inclusions were mainly skein-like (Fig. 1b) or dot-like cytoplasmic inclusions in the reticular formation and/or the inferior olivary nucleus. In some cases, pTDP-43 pathology was also observed in the XII nerve nucleus. The pTDP-43 pathology in the sensory cortex was similar to that of the motor cortex, but with a lesser degree of severity. In the hippocampus (Fig. 1c), there was marked involvement of the granule cells of the dentate gyrus, which in some cases was accompanied by inclusions in neurons of the CA regions.

The distribution of ALS cases across the neuropathological stages is outlined in Table 2: one case had Stage 1 (motor cortex pathology only), 8 had Stage 2 (Stage 1 + brainstem pathology), 16 had Stage 3 (Stage 2 + sensory or striatal pathology), and 9 had Stage 4 (Stage 3 + hippocampal pathology in 6, and hippocampal pathology in 3). Of these 34 cases, four had slightly variant distributions of pTDP-43 pathology: two cases fit the staging scheme only after review of the striatum as an alternate region to the sensory cortex, one was included in Stage 2 with pTDP-43 immunopositivity in the brainstem but none in the motor cortex due to a complete loss of Betz cells in the section examined, and one was included in Stage 3 with pTDP-43 immunopositivity in the motor and sensory cortices but none in the brainstem, due to a lack of available tissue at the appropriate level. Inherently, all of our cases fit the neuropathological staging scheme suggesting that the staging scheme can successfully be applied to typical ALS cohorts and that the ALS cases assessed in this investigation were comparable with previous studies.

### Assessment of motor cortex severity and oligodendroglial involvement

The severity of Betz cell loss was variable across all stages and did not influence the severity of pTDP-43 pathology in the motor cortex. Correlations revealed no relationship between the loss of Betz cells and disease stage or the severity of pTDP-43 pathology (Additional file 2: Figure S2). These data show that the absence of pTDP-43 pathology in the posterior limb of the internal capsule of all cases is not due to a complete loss of motor neurons from all cases, and particularly from cases with Stage 4 disease (see Additional file 2: Figure S2). Furthermore, the density of oligodendrocytes in ALS did not appear to differ from that seen in control sections (see Additional file 3: Figure S3) suggesting a loss of oligodendrocytes does not underlie the absence of pTDP-43 staining.

**Fig. 2:**
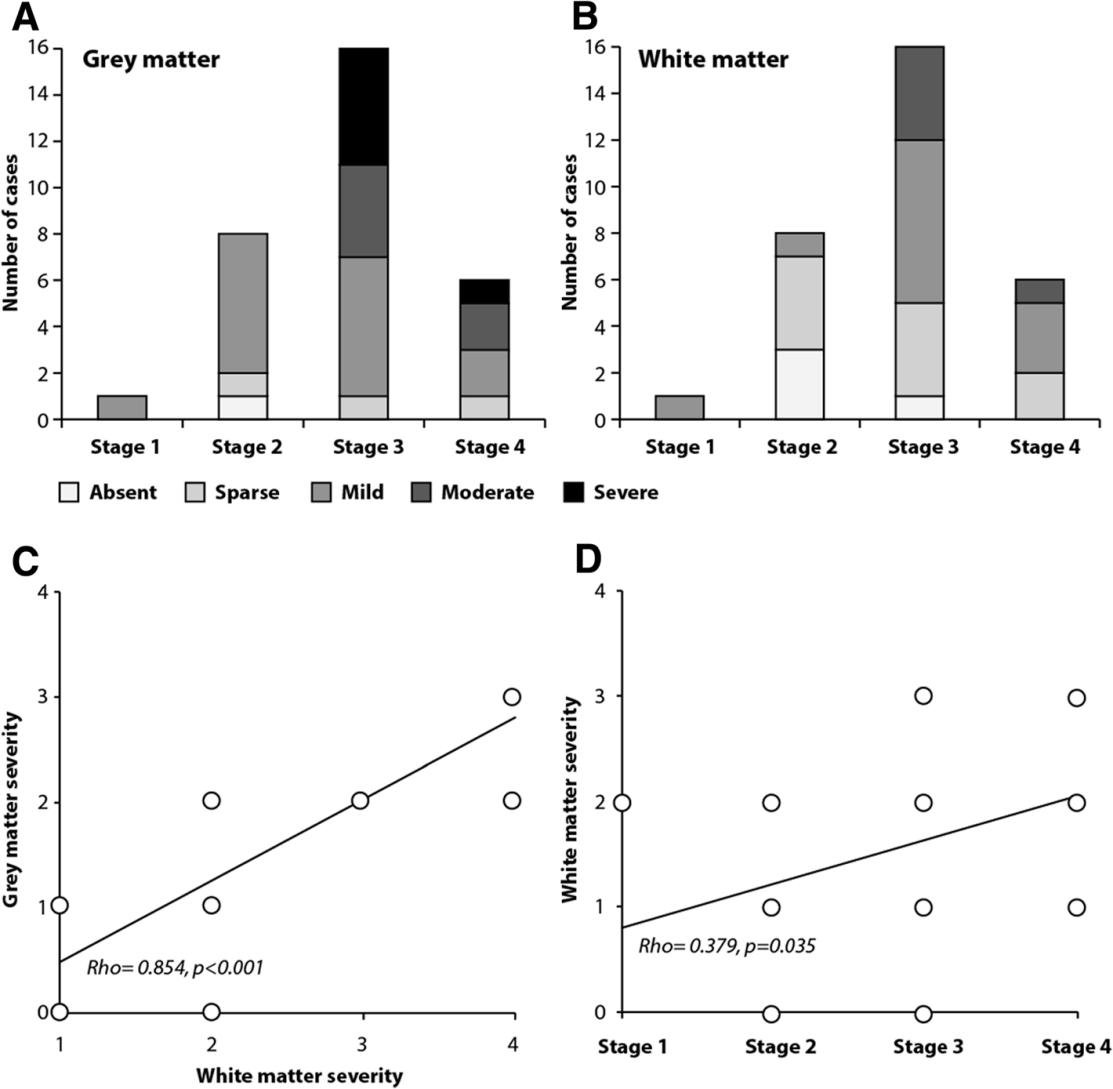
The proportion of cases with increasing severity of (**a**) grey and (**b**) white matter pTDP-43 pathology across the disease stages, (**c**) linear relationship between the severity of pTDP-43 pathology in the grey matter and white matter, and (**d**) linear relationship between the severity of pTDP-43 pathology in the white matter and disease stage

The severity of pathology in the motor cortex (Fig. 2a) and its subcortical white matter (Fig. 2b) was graded semi-quantitatively, and the distribution of severity grades across the four stages plotted. Moderate to severe pTDP-43 pathology was only observed in Stages 3 and 4. Correlation analysis showed that the severity of pTDP-43 pathology in the underlying white matter was linearly associated to that in the overlying grey matter (Rho = 0.854, *p* < 0.001) with the grey matter having more severe pTDP-43 pathology on average (Fig. 2c). A significant correlation was found between the severity of pTDP-43 pathology in the white matter and neuropathological stage (Rho = 0.379, *p* = 0.035, Fig. 2d), with Stage 1 included or not in the analysis. In addition, there was a trend for the severity of pTDP-43 pathology in the grey matter severity to correlate to neuropathological stage (Rho = 0.349 *p* = 0.055). While the severity of pTDP-43 pathology in the motor cortex and the underlying white matter did appear to increase with stage, the stages themselves did not appear to reflect the same clinical course of disease for all groups (see below).

**Fig. 3:**
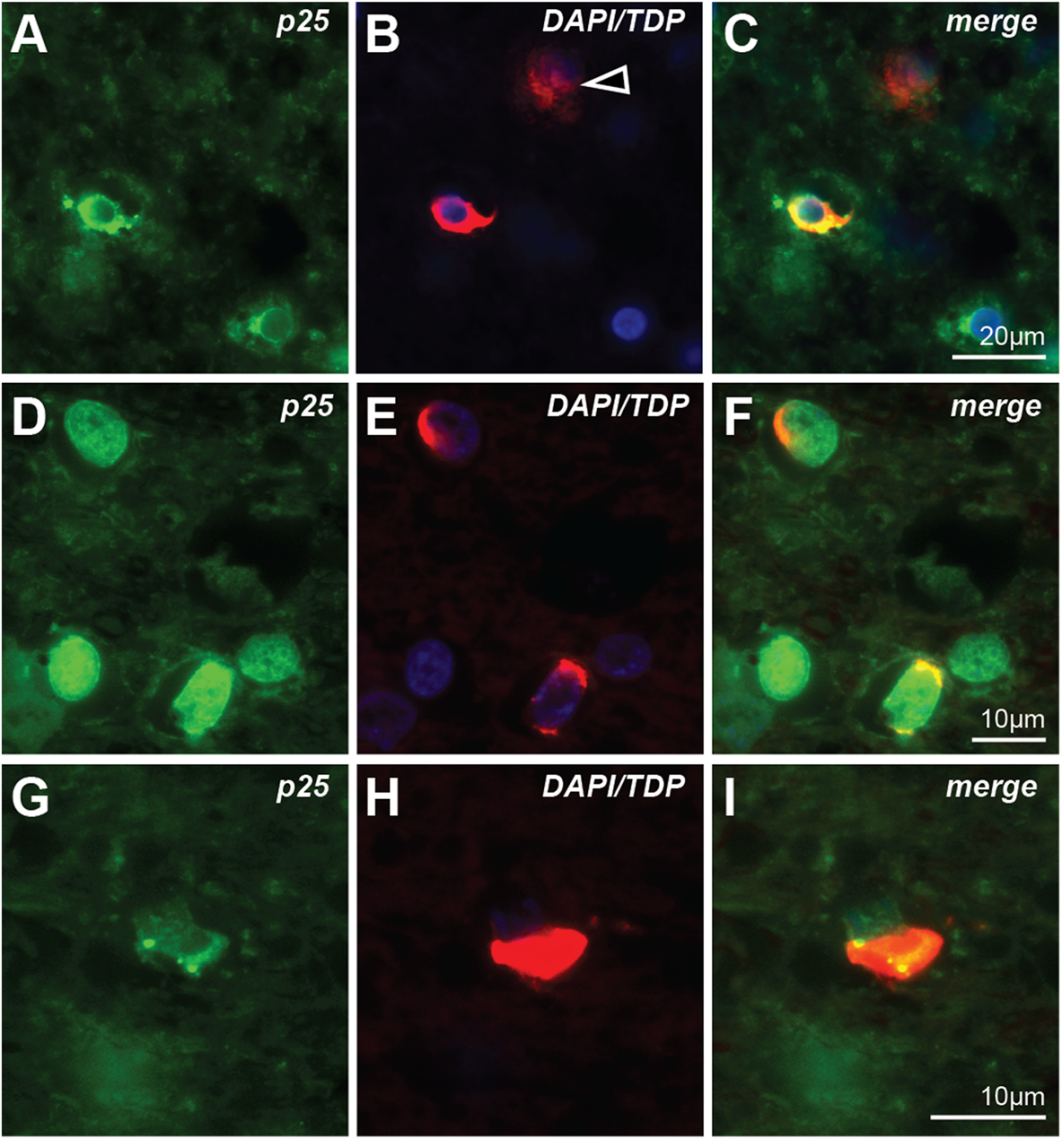
Co-localisation of pTDP-43 pathologies (red) in oligodendrocytes (green) and neurons (arrowhead) in the motor cortex (**a**-**c**) and the underlying white matter (**d**-**i**)

**Fig. 4:**
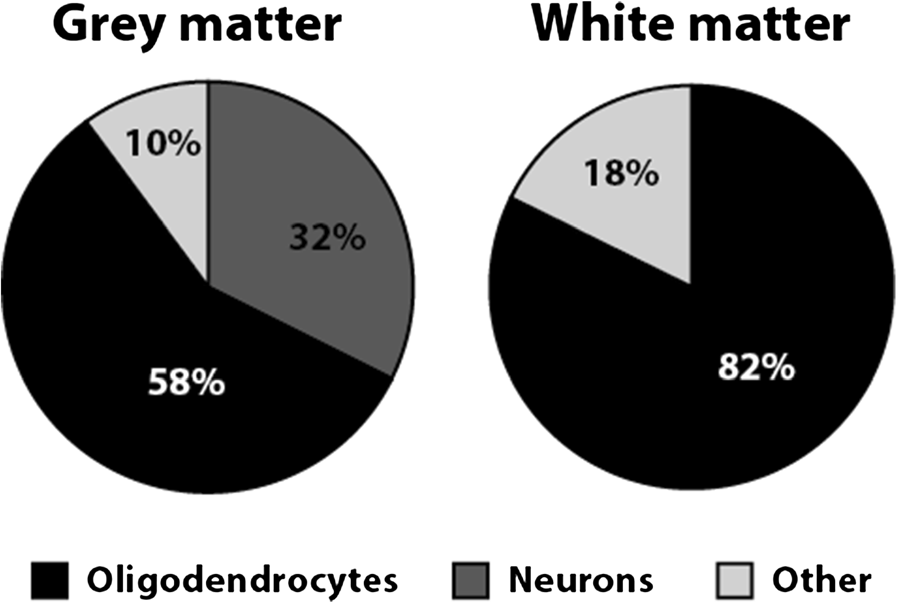
Distribution of grey and white matter pathology in the motor cortex. Phosphorylated TDP-43 inclusions were observed in either neurons, oligodendrocytes or “other” non-p25α immunoreactive glial cells with an obvious nucleus, potential p25α immunoreactive pTDP-43 extra-axonal inclusions (not associated with a nucleus), or pTDP-43 inclusions detached from any other identifiable structures, such as dystrophic neurites

For the cases with severe grey matter pathology (*n* = 6), double-labelling immunofluorescence was performed to confirm that the pTDP-43-immunoreactive aggregates observed in the motor cortex and underlying white matter were indeed mainly in neurons and oligodendrocytes. Double labelling with the oligodendrocyte specific p25α antibody showed pTDP-43-immunoreactive inclusions either surrounding oligodendroglial nuclei (Fig. 3a-f) or wrapped around supposed oligodendrocyte-ensheathed axons (Fig. 3g-i). Neuronal pTDP-43-immunoreactive inclusions (Fig. 3b arrowhead) were more diffuse, consistent with the pathology observed with pTDP-43 immunoperoxidase staining. Quantitation showed that the majority of inclusions (58 % in the grey matter) were found in oligodendrocytes (Fig. 4), with a relative absence of inclusions observed in satellite oligodendrocytes (Additional file 4: Figure S4).

### Relationship between pathological staging and clinical progression

As only one case had Stage 1 disease, this group was excluded prior to nonparametric analyses. Group analyses between pathological stages and clinical characteristics for all other cases are summarised in Table 3. A similar age at onset might be expected in sporadic ALS cases of different stages, however only cases in Stages 2 and 3 had a similar age at disease onset, while cases with Stage 4 presented approximately 10 years later (Table 3). Progressively longer disease duration might also be expected if there is progression from one stage to the next, however, while Stage 3 had a longer disease duration than Stage 2, Stage 4 did not differ from Stage 2 (Table 3). Since Stages 2 and 3 presented with a similar age at onset, the longer disease duration in Stage 3 is consistent with these two groups being on a continuum. Stage 4 however, presented with later-onset disease and had a shorter disease duration than Stage 3, suggesting that this group does not lie on the same continuum and possibly represents a separate ALS cohort with an altered disease course (Additional file 5: Figure S5).

**Table 3:** Relationship between pathological staging and clinical progression

	Stage 2	Stage 3	Stage 4	p-value
Gender (M:F)	5:3	10:6	6:3	0.628^a^
Mean age at onset (SD)	60 (9)	58 (6)	70 (8)*	0.007^b^
Mean age at death (SD)	61 (9)	61 (6)	71 (8)*	0.017^b^
Mean duration since diagnosis (SD)	1.5 (0.7)	3.5 (1.7)*	1.4 (0.6)	0.002^b^
Bulbar onset (N)	3	4	2	0.904^a^
Upper limb onset (N)	2	6	3	
Lower limb onset (N)	3	6	4	

shading indicates p < 0.05

^a^χ^2^ analysis

^b^Kruskall Wallis test

*significantly different group using posthoc Mann–Whitney *U* test

## Discussion

While conventionally regarded to be a disease that exclusively targets the motor system network, imaging and pathological studies have since provided evidence to suggest that ALS is a multisystem neurodegenerative disorder. This can be seen by the presence of characteristic pTDP-43 deposits in brain regions outside the pyramidal motor system [5–7, 23], and the demonstration of extramotor involvement using neuroimaging techniques [8–10] The progression of ALS throughout the brain has been staged using two different modalities- pathological spread using autopsy samples [6] and spread through white matter tracts using diffusion tensor imaging [8]. Currently, the concept of disease progression in ALS involves a theory of corticofugal axonal spread, suggesting that grey matter regions become sequentially involved through the white matter tracts that connect them [7].

In this study, grey matter immunostaining revealed cytoplasmic pTDP-43 inclusions in the neurons and glia of the motor cortex, brainstem, sensory cortex, striatum and hippocampus confirming previous studies reporting deposits in areas within and outside the motor system [5–7, 23] and independently validating the ALS neuropathological staging scheme recently proposed by Brettschneider and colleagues [6]. Corresponding white matter immunostaining revealed glial pTDP-43-immunoreactive pathology immediately under the motor and sensory cortices. Double-labeling immunofluorescence confirmed that glial pathology observed in both grey and white matter was co-localised in oligodendrocytes. Thus our cases appear to be consistent with those reported in the literature.

In the present study we sought evidence for transmission along axonal pathways by looking for similar pTDP-43 oligodendrocyte pathology in the posterior limb of the internal capsule, the corpus callosum and the cingulum bundle. Alterations to diffusion parameters are believed to reflect pathophysiological damage to axons and myelin [24, 25], suggesting inclusions in oligodendrocytes might be found along these tracts. Contrary to this, p-TDP-43 immunostaining revealed no such evidence. This was an unexpected result given the prominence of these white matter tracts in ALS diffusion imaging. For instance, reductions in fractional anisotropy and changes to mean diffusivity present as consistent findings in the internal capsule [8–10], with changes more pronounced in the posterior limb through which fibres descend from the direct corticospinal tract [26]. The corpus callosum and cingulum bundle have similarly been identified as involved white matter regions, even across different phenotypes of ALS and in those with little clinical upper motor neuron involvement [8–10]. A pathological study has shown that 30 % of 27 ALS cases had white matter pTDP-43-immunoreactivity under the cingulate cortex (median severity score of 0.4 on a scale of 0 (none) to 3 (severe)) [23] and so a lack of pTDP-43 pathology in the cingulum bundle in the present study was unexpected, especially considering its connection to areas reportedly involved by end stage disease [6, 7]. In the motor region, the severity of white matter pTDP-43 pathology linearly associated with the severity of pathology in the grey matter, consistent with similar data from the spinal cord [14] where the severity of pTDP-43 pathology and neuronal loss correlated closely with grey and white matter oligodendrocyte involvement, and to the onset of disease in the spinal cord. Spinal cord oligodendrocyte involvement was reported to occur in areas otherwise void of pTDP-43 aggregates and neuronal loss, suggesting that grey matter oligodendroglial involvement represents an early event in the disease process [14].

Our data revealed oligodendrocyte pTDP-43 pathology close to involved cortical areas and not further along axonal tracts, suggesting a more localised mechanism of pTDP-43 spread. Reports on intra-axonal pTDP-43 aggregates remain limited to the lower motor neurons of the brainstem and spinal cord [6, 7, 14]. An assessment of Betz cells revealed no relationship between the number of surviving Betz cells and the grey matter severity score, confirming that the lack of deep white matter pTDP-43 pathology in regions such as the posterior limb of the internal capsule was not due to the loss of upper motor neurons and degeneration of their axons. Given that healthy Betz cells require support from functional oligodendrocytes, it is further unlikely that the lack of oligodendrocytic pathology was due to a loss of oligodendroglia, a suggestion supported by our finding of a similar density of oligodendrocytes in white matter tracts of ALS and controls (Additional file 3: Figure S3).

Our data provide some evidence that the early pathological stages of ALS may be on a continuum based on their mean age at onset and increasing disease duration (Additional file 5: Figure S5). However, the ALS cases with the greatest spread of pathology (Stage 4) were significantly older at onset and had short disease duration, suggesting a difference between this group and other disease stages. Further exploration of these findings is warranted, especially in ALS cases with co-existing dementia as such cases were excluded from this study.

## Conclusions

The present study validates the neuropathological staging scheme and confirms that regional assessment of pTDP-43 pathology allows for the identification of four specific stages as described previously [6]. Our data offer support to the suggestion that Stages 2 and 3 sit on a continuum, while Stage 4 may represent an alternate phenotype as this group is significantly older at onset and has a shorter disease duration. Despite a predominance of oligodendroglial pTDP pathology in the grey matter regions affected and evidence of affected Betz cell in many cases, no case had observable pTDP-43 oligodendrocyte pathology in remote but connected white matter tracts. Considering the careful selection of these tracts from neuroimaging evidence of the progression of ALS pathology along them, the lack of pTDP-43 pathology in deep white matter tracts questions the concept that pTDP-43 pathology underlies these neuroimaging changes. Further clarification is required to determine the cellular correlates of the white matter changes identified in ALS, as involvement of these tracts is not related to significant pTDP-43 pathology.

## Additional files

Additional file 1: Figure S1. Examples of the semi-quantitative scoring for pTDP-43 immunoperoxidase pathology in the motor cortex (A-D) and underlying white matter (E-G): sparse in cortex (A) and white matter (E); mild in cortex (B) and white matter (F); moderate in cortex (C) and white matter (G); severe in cortex (D). Double labelling immunofluorescence confirmed that most white matter inclusions were localised in oligodendrocytes (H). Scale bar in G applies to all figures. (PNG 2663 kb) Additional file 2: Figure S2. Absence of any relationship between Betz cell numbers and either disease stage (A) or the severity of grey matter pTDP-43 pathology (B) (EPS 732 kb) Additional file 3: Figure S3. There was no obvious difference in the density of oligodendrocytes in sections of the posterior limb of the internal capsule (A), the corpus callosum (B, C) or the cingulum (D, E) between controls (B, D) and ALS cases (A, C, E) Scale in E applies to B-D. (TIFF 4930 kb) Additional file 4: Figure S4. Assessment of affected neurons in the motor cortex revealed limited oligodendrocytic pTDP-43 pathology in satellite oligodendrocytes (C, arrowheads), even when neurons contained pTDP-43 immunorectivity (A-C). Pathologically affected oligodendrocytes were generally at some distance from affected neurons (D), Scale bar in B applies to C and D. (TIFF 9184 kb) Additional file 5: Figure S5. Stylised differences in age at onset and disease duration between the neuropathological stages in the cohort suggests that Stage 4 does not lie on the same continuum as Stages 2 and 3 (PNG 6 kb)

## Abbreviations

ALS: Amyotrophic lateral sclerosis
pTDP-43: Phosphorylated 43-kDA TAR DNA-binding protein
