# Supplementary figures and images for: Spread of pathology in amyotrophic lateral sclerosis: assessment of phosphorylated TDP-43 along axonal pathways
Source: Acta Neuropathol Commun. 2015 Jul 28;3:47. doi: 10.1186/s40478-015-0226-y (PMC4517552; doi:10.1186/s40478-015-0226-y)

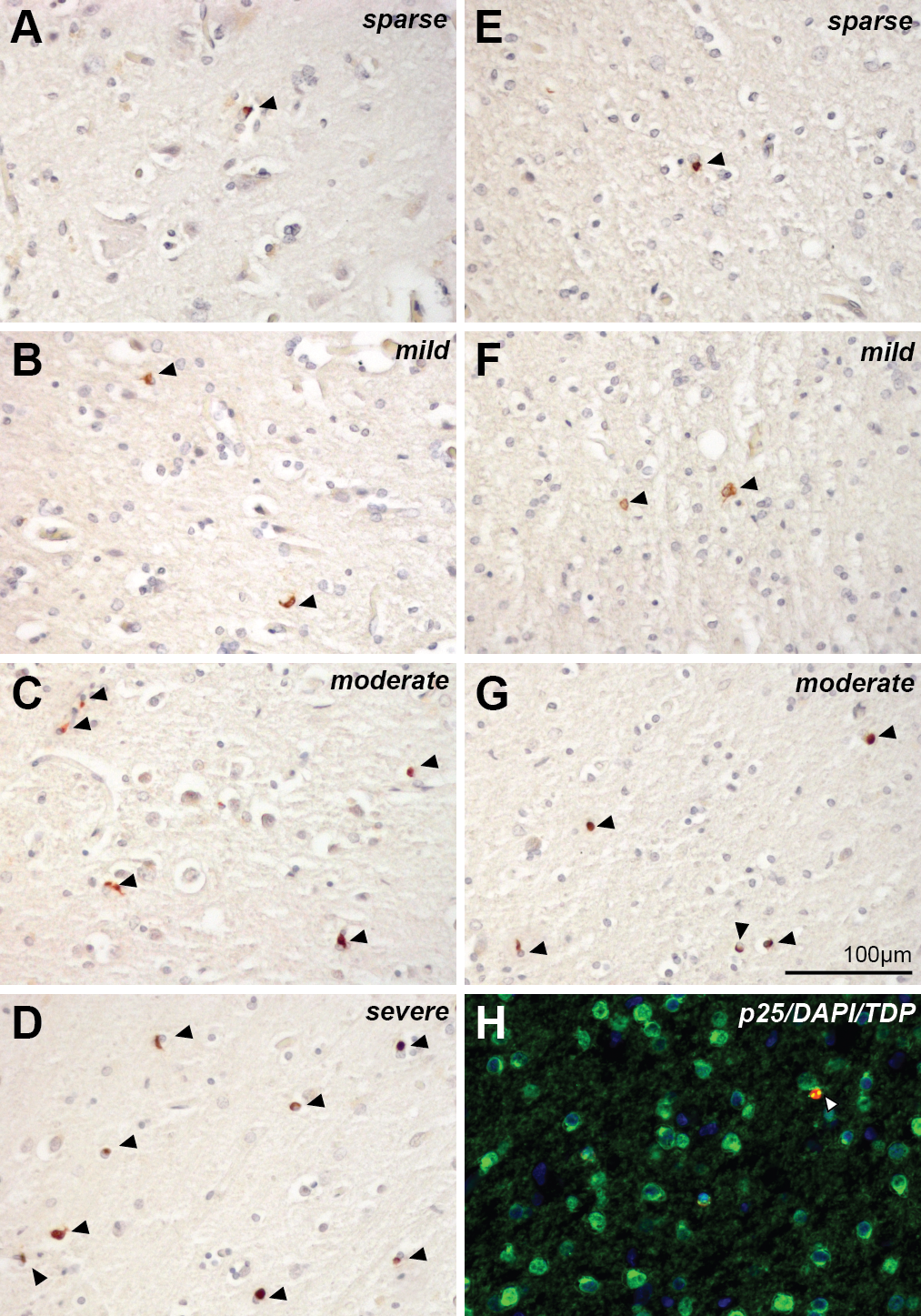

Supplement: Additional file 1: Figure S1. — Examples of the semi-quantitative scoring for pTDP-43 immunoperoxidase pathology in the motor cortex (A-D) and underlying white matter (E-G): sparse in cortex (A) and white matter (E); mild in cortex (B) and white matter (F); moderate in cortex (C) and white matter (G); severe in cortex (D). Double labelling immunofluorescence confirmed that most white matter inclusions were localised in oligodendrocytes (H). Scale bar in G applies to all figures. (PNG 2663 kb) [file 40478_2015_226_MOESM1_ESM.png]

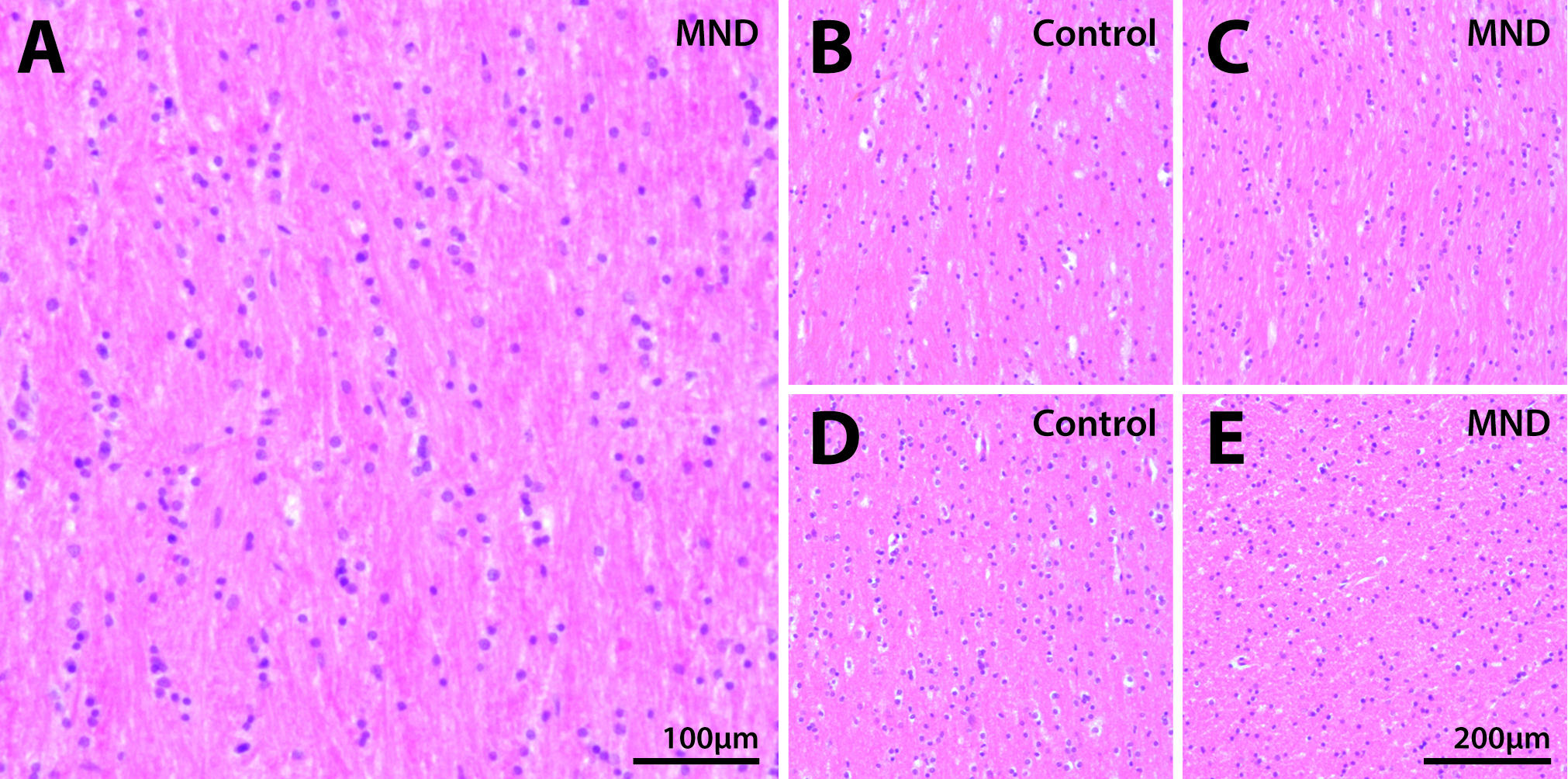

Supplement: Additional file 3: Figure S3. — There was no obvious difference in the density of oligodendrocytes in sections of the posterior limb of the internal capsule (A), the corpus callosum (B, C) or the cingulum (D, E) between controls (B, D) and ALS cases (A, C, E) Scale in E applies to B-D. (TIFF 4930 kb) [file 40478_2015_226_MOESM3_ESM.tif]

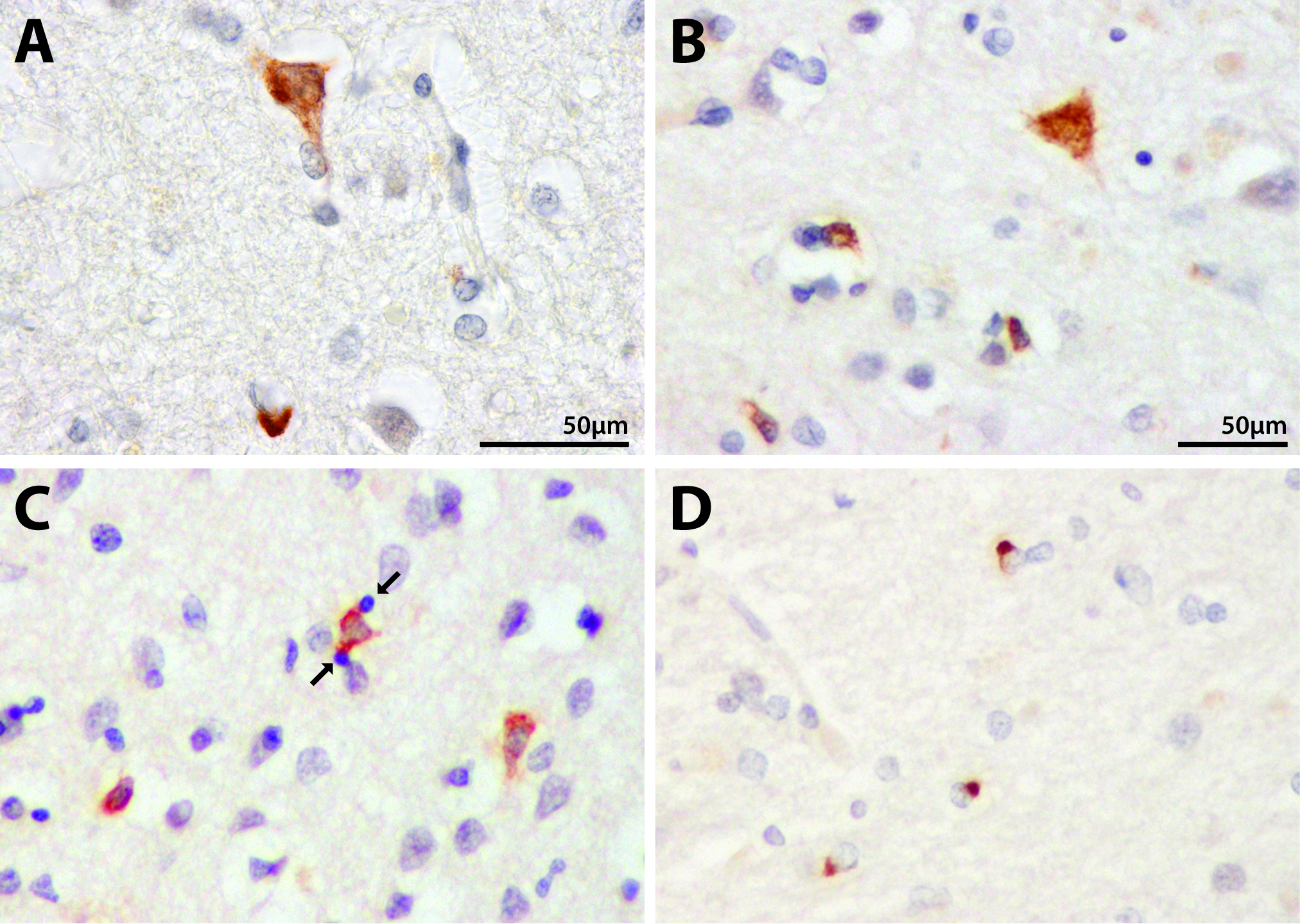

Supplement: Additional file 4: Figure S4. — Assessment of affected neurons in the motor cortex revealed limited oligodendrocytic pTDP-43 pathology in satellite oligodendrocytes (C, arrowheads), even when neurons contained pTDP-43 immunorectivity (A-C). Pathologically affected oligodendrocytes were generally at some distance from affected neurons (D), Scale bar in B applies to C and D. (TIFF 9184 kb) [file 40478_2015_226_MOESM4_ESM.tif]

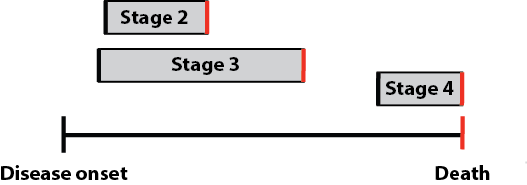

Supplement: Additional file 5: Figure S5. — Stylised differences in age at onset and disease duration between the neuropathological stages in the cohort suggests that Stage 4 does not lie on the same continuum as Stages 2 and 3 (PNG 6 kb) [file 40478_2015_226_MOESM5_ESM.png]
